# Quantitative assessment of visual cortex function with fMRI at 7 Tesla—test–retest variability

**DOI:** 10.3389/fnhum.2015.00477

**Published:** 2015-09-01

**Authors:** Aini Ismafairus Abd Hamid, Oliver Speck, Michael B. Hoffmann

**Affiliations:** ^1^Department of Biomedical Magnetic Resonance, Institute for Experimental Physics, Otto-von-Guericke University MagdeburgMagdeburg, Germany; ^2^Department of Neurosciences, School of Medical Sciences Health Campus, Universiti Sains MalaysiaKubang Kerian, Malaysia; ^3^Center for Behavioral Brain SciencesMagdeburg, Germany; ^4^Leibniz Institute for NeurobiologyMagdeburg, Germany; ^5^German Center for Neurodegenerative DiseaseMagdeburg, Germany; ^6^Visual Processing Laboratory, Department of Ophthalmology, Otto-von-Guericke University MagdeburgMagdeburg, Germany

**Keywords:** test-retest, longitudinal, reliability, reproducibility, retinotopic maps, fMRI, visual areas

## Abstract

fMRI-based retinotopic mapping was used to assess systematic variations in activated cortical surface area, amplitude, and coherence across sessions. Seven healthy subjects were scanned at 7 T in three separate sessions with intervals of 51.4 ± 5.4 days (Sessions 1 and 2) and 167.9 ± 24.4 days (Sessions 2 and 3). We found a reduction between Sessions 1 and 2 for activated cortical surface area, between Sessions 1 and 3 for amplitude, and between Sessions 1 and 2/3 for coherence. The results do not support head motion as a major cause of the observed effect seen in Session 1, suggesting that cognitive effects were the underlying cause of change. The phase correlations for both eccentricity and polar angle mapping were highly correlated between sessions, demonstrating the stability of the maps. Furthermore, the sensitivity in determining inter-session changes of cortical surface area, response amplitude, and coherence were, at a 5% significance level, estimated to be 1.5, 6, and 5%, respectively. Any future longitudinal fMRI study should carefully evaluate activation across sessions to determine the eligibility of inclusion of all time points. This experimental design provides guidance in methodological issues of clinical longitudinal fMRI-studies, specifically regarding effects of subject experience.

## Introduction

Functional magnetic resonance imaging (fMRI) is a widely used method for investigating human brain functions. Retinotopic mapping is the gold standard to define visual field locations in the occipital cortex. Recent advances in fMRI using retinotopic mapping stimuli enable the identification of individual cortical visual areas and the functional specialization of the visual system. Besides fMRI, positron emission tomography (PET), and event-related potentials (ERPs) can also be used to identify visual topography or retinotopic organization in the human visual cortex (Woldorff et al., 1997). There are a number of factors that influence fMRI results; these factors can stem from the image signal-to-noise ratio, motion artifacts, magnetic field inhomogeneities, cognitive processes, cognitive strategies over time, or other causes (Krüger and Glover, 2001; Huettel et al., 2008; Bennett and Miller, 2010).

Test-retest studies have been used in the field of neuroscience to understand how response variabilities occur over time and influence detection sensitivity. In clinical studies of neurological disorders, for example, repeated measures have the potential to identify changes in brain activation, structure, and connectivity associated with disease progression, treatment or rehabilitation. To understand treatment effects, knowledge of the underlying test–retest variability for repeated measures of normal subjects' brain activation patterns is essential. Test-retest studies of normal subjects can provide guidance for the consideration of experience-driven effects and other methodological issues (Kelly et al., 2006).

Previous studies have investigated fMRI test–retest results from a wide range of tasks relevant to auditory, visual, motor, and cognitive stimuli (Specht et al., 2003; Johnstone et al., 2005; Kong et al., 2007; Raemaekers et al., 2007; Friedman et al., 2008; Caceres et al., 2009; Bressler and Silver, 2010; Seibert et al., 2012). For the visual areas and retinotopic mapping itself, however, there is only little knowledge. Bressler and Silver (2010) used a rotating checkerboard wedges stimulus, and the subjects were instructed to direct their attention to either the wedge (attention-to-wedges task) or the central fixation point (attention-to-fixation task). They reported that spatial attention tasks improve the reliability of fMRI retinotopic mapping signals in the occipital and parietal cortex.

Test-retest reliability of fMRI results has been assessed in many ways, for example using the Pearson product-moment correlation coefficient (Pearsons's *r*; Fernández et al., 2003), maximum likelihood (Maitra et al., 2002), or intraclass correlation coefficient (Raemaekers et al., 2007; Friedman et al., 2008; Caceres et al., 2009; Bressler and Silver, 2010; Seibert et al., 2012). To our knowledge no gold standard exists. In this study we aim to assess systematic variations in the fMRI responses between sessions. Therefore, we have applied a number of previously used measures to compare the activation between sessions. In order to make our work comparable to previous studies, we have included activation extent, response amplitude and coherence. Additionally, to evaluate the similarity of the obtained maps across sessions, we examined the reproducibility of the phase maps in the early visual areas.

Although retinotopic mapping has been a common and widely used method for visual studies, there is only limited knowledge on within-subject changes of brain activity, specifically in test–retest paradigms. In this study, conventional retinotopic mapping, i.e., eccentricity and polar angle mapping, and full field tasks were used to assess systematic variations in activated cortical surface area, fMRI signal amplitude, and coherence across sessions.

## Materials and methods

### Subjects

Seven subjects (3 female; age 29 ± 4 years), with no history of ophthalmological or neurological disease, participated in the study. The procedures followed the tenets of the declaration of Helsinki (World Medical Association, 2000) and the protocol was approved by the ethics committee of the University of Magdeburg, Germany. The subjects gave written and informed consent. The test–retest experiment took place at the same time of day with intervals of 51 ± 5 days (Sessions 1 and 2) and 168 ± 24 days (Sessions 2 and 3). Two of the subjects involved were unfamiliar with the procedure (subjects 1 and 7), the other five were experienced fMRI subjects including one who had participated in a different retinotopic mapping study (subject 6) 10 months prior to session 1 of this study (see Supplementary information for the individual result patterns).

### Visual stimulation for fMRI

In order to avoid differential order effects of visual stimulation conditions across subjects, each of the three fMRI sessions comprised the same sequence of experiments: eccentricity mapping with (a) expanding and (b) contracting rings (scan 1 and 3); polar-angle mapping with rotating wedges (a) counter-clockwise and (b) clockwise (scan 2 and 4); and block design full-field stimulation starting with (a) stimulation on and then (b) stimulation off (scan 5 and 6). The visual stimuli were back projected with a video projector (DLA-G150CL, JVC Ltd.) onto a screen (horizontal viewing angle ~14.8° and vertical viewing angle ~8.3°) behind the subject and viewed via a surface mirror mounted onto the head coil. The stimulus patterns were approximately m-scaled to compensate for the cortical magnification of the visual stimulus (width of the most central and most peripheral ring 0.1° and 1.0°) and comprised a contrast inverting (with 8 reversals per second) circular checkerboard of 7.4° radius consisting of black and white checks with 24 segments and 26 rings (mean luminance 62 cd/m^2^, contrast 99%). Identical presentation of the visual stimulation is necessary to allow comparability between the three-time points. The average stimulus luminance of 62 cd/m^2^ and 99% contrast were ensured at all three points based on photometer calibration (CS-100A Konica Minolta). To produce the desired luminance and contrast of the stimuli, the gamma function, which relates numeric input and visual output for each projector color channel, has been considered in the computed RGB-values.

The subjects were instructed to fixate on a central-fixation marker (0.34° diameter) and to report any color changes (200 ms duration at intervals between 5 and 10 s) by pressing a button. Each experimental scan started with a 12 s scan dummy stimulation period and was followed by seven 36 s cycles of visual stimulation for rings and rotating wedges and ten 24 s cycles of visual stimulation for full field. Phase-encoded paradigms in retinotopic mapping stimulated different parts of the visual field during different stimulation epochs, i.e., increasing or decreasing polar angle for (i) polar-angle mapping and increasing or decreasing eccentricities for (ii) eccentricity mapping. Thus, only a section of the contrast-reversing checkerboard was presented at a time: (i) Polar angle mapping: Polar angle stimuli stepping through clockwise and counterclockwise direction were presented. The wedge was six segments (90°) wide and stepped 54 times by 6.7° in each of the 36 s cycles. (ii) Eccentricity mapping: The stimulus moved through the eccentricities as an expanding or contracting ring. The ring was 0.97° wide and stepped 54 times by 0.32° in each of the cycles. It expanded uniformly beyond the maximum extent of the screen for 5 s and then wrapped around to the fovea. This stimulation gap helped to distinguish peripheral from fovea responses in the eccentricity mapping data (Hoffmann et al., 2009). For the third type of visual stimulation, i.e., full field stimulation, subjects viewed 12 s checkerboard reversal (8 reversals per second) followed by 12 s gray (same mean luminance in both epochs) in a block design. Altogether, the fMRI measurements took around 30 min.

### fMRI acquisition

For functional imaging, T2^*^-weighted echo-planar images (EPI) were acquired with a 7 Tesla whole body MRI scanner (Siemens Healthcare, Erlangen, Germany) using a 32 channel head coil (Nova Medical, Wilmington, MA). The gradient-echo EPI pulse sequence had the following parameters: repetition time (TR) = 3000 ms, echo time (TE) = 21 ms, field of view (FOV) = 140 mm, flip angle (α) = 90, and voxel size = 1 × 1 × 1 mm. Forty-five slices were acquired perpendicular to the calcarine sulcus for a duration 264 s with 88 volumes (dummy stimulation period and 7 cycles) for eccentricity and polar angle mapping, and 252 s with 84 volumes (dummy stimulation period and 10 cycles) for the full-field stimulation. In addition, high-resolution anatomical images of the occipital region were obtained using T1-weighted 3D-MPRAGE with the following parameters: acquisition time = 296 s, TR = 2300 ms, TE = 2.66 ms, inversion time = 1050 ms, FOV = 256 mm, isotropic resolution = 1 mm. 3D-MPRAGE inhomogeneity was corrected by division with 3D gradient echo (GE) reference data (Van de Moortele et al., 2009) without inversion and otherwise identical parameters but: acquisition time = 170 s, TR = 1340 ms. The functional images were corrected online for motion and spatial distortions (In and Speck, 2012).

Over the course of all the experiments, no MR system upgrade or modification took place and no auxiliary equipment had been installed or removed. The MR system performance for fMRI experiments has been tested at least once per week as part of the regular quality assurance procedure and no relevant parameters (SNR, signal stability, signal homogeneity, RF-parameters, EPI ghost level) showed any deviation from the established reference values.

### Cortical flattening

In order to provide homogenous white and gray matter contrast for segmentation, T1-weighted inhomogeneity corrected MPRAGE images (Van de Moortele et al., 2009) were used to segment the cortex and to create a flattened representation of the cortical gray matter (Teo et al., 1997; Wandell et al., 2000). Gray and white matter were segmented using Freesurfer, ITK Gray, 1.6.0.1., and mrGray (VISTA) and were manually edited to minimize segmentation errors. The anatomical segmentation and flattening was done once per subject.

### Preprocessing of functional images

In the three scanning sessions, identical methods were used for fMRI data analysis. The functional images were analyzed using the Stanford VISTA-Tools (VISTA; http://white.stanford.edu/software/). The T2^*^-weighted images were aligned to the T1-weighted images' coordinate frame to project the fMRI time series onto the flattened cortex representation. Each voxel's time series (TS) underwent the following analysis steps: (1) dummy stimulation period (12 s or 4 volumes) of each functional run was discarded to avoid transient-onset artifact; (2) final single cycle (36 s, i.e., 12 volumes) at the end of each functional run was also discarded for eccentricity and polar angle due to a reconstruction failure and an incorrect volume setting for two subjects in one of the sessions. These two scans were not excluded because only a few volumes at the end of the scans were involved. Thus, six stimulation cycles were retained for eccentricity and polar angle mapping and 10 stimulation cycles for full-field analysis for each subject; (3) TS were divided by their mean intensity; (4) for one of the opposing directions in the three types of visual stimulation (contracting vs. expanding rings, clockwise vs. counter clockwise rotating wedges, and on vs. off start of full field stimulation) the TS were flipped in time. Then the hemodynamic delay of the flipped TS was corrected by shifting the TS by 6 s (i.e., two TR). (5) TS of opposing directions were averaged to remove or cancel the residual phase lags caused by residual hemodynamic delays of the two stimulus directions. This technique allows examining the true phase of the eccentricity and polar angle (Sereno et al., 1995; Smith et al., 1998; Silver et al., 2005; Larsson and Heeger, 2006); (6) data were Fourier transformed to calculate the amplitude and the response phase for the stimulus frequency; (7) the correlation, technically coherence, was measured (Engel, 1997). The coherence is calculated from the time series with a sinusoid with the frequency having the same value as the fundamental frequency (i.e., 1/36 Hz for 36 s stimulus described above) of the visual stimulation (Engel, 1997). The coherence value was determined independently for each voxel in the functional scan. The coherence value represents the strength of the response of that voxels to each visual stimulation condition.

For qualitative assessment and presentation of the response maps in the flattened representation, the coherence and phase values were smoothed by convolving a Gaussian Kernel (full width at half maximum: 4 mm) with the complex-vector representation of the BOLD response. The statistical significance levels, or *p*-values, for associated coherence values were estimated according to Silver et al. (2005). For all repeated measures of individual results, a threshold of *p* = 0.05 was applied for multiple comparison (Zandbelt et al., 2008; Plichta et al., 2012): only voxels with coherence values exceeding 0.18 were considered for further analysis. No spatial or temporal filtering was applied for any quantitative analyses (amplitude, coherence, or any responses in the EPI planes) to avoid loss of resolution. Data quality evaluation was performed through direct visual inspection and did not show signal dropouts due to local susceptibility artifact.

The identification of the visual areas V1, V2, and V3 was performed on the data averaged across all three sessions in order to avoid the intrusion of systematic inter-session effects. The boundaries between the visual areas were delineated manually on the cortical flat maps based on the response phase reflected in the false-color maps for polar angle and eccentricity (Engel et al., 1994; Engel, 1997; Wandell et al., 2007). The borders of the visual areas were representations of the vertical and horizontal meridians in the visual field according to established procedures (Wandell et al., 2007). Subsequently, visual area definitions were used to examine the correlation of phase maps and fMRI signal difference between sessions (Swisher et al., 2007).

### Head motion assessment

Head motion during the fMRI acquisitions was quantified using the uncorrected functional images, i.e., without the online motion and distortion correction. For the relevant volumes (72 volumes for eccentricity and polar angle, 80 volumes for full field), head motion was quantified for each scan with McFlirt version 5.0 [motion correction using FMRIB's Linear Image Registration Tool (Jenkinson et al., 2002)] and FSL (FMRIB's Software Library, www.fmrib.ox.ac.uk/fsl). The head motion metrics were evaluated from three translation (x, y, and z in mm) and three rotation (x, y, and z in rad) parameters. Then, a head motion index (in mm) was calculated by the root mean square (RMS) of the six parameters (three rotations and three translations) (Silver et al., 2005).

### Statistical analysis

First, in order to explain the data variance and assign it to the different possible sources (e.g., brain areas and condition), we investigated the variability or change in activated cortical surface area, amplitude, and coherence using repeated measures analysis of variance (ANOVA) by session as a within-subject factor, and visual area and stimulation condition as the between-subject factors. Because the coherence values were not normally distributed, they were converted into normally distributed values by Fisher transformation for further statistical analysis. In this study, *post-hoc* test Sidak correction was used to account for multiple testing to detail the factor effect. Second, we assessed the minimal effect sizes to be detected with the current design for 95% confidence intervals. Third, in order to evaluate the similarity of the obtained maps across sessions, we examined the reproducibility of the phase maps in the early visual areas using circular correlation [circular Statistic toolbox in Matlab as suggested by Philipp (2009)]. The resulting correlations were converted by Fisher's z-transformation for further statistical analysis. Quantitative comparison of phase correlation was determined separately for each visual area (V1, V2, and V3) and visual stimulation (eccentricity and polar angle) using independent *t*-tests. Additionally, we evaluated the change in head motion across sessions by repeated measures ANOVA.

## Results

### Bold signals

**Figure 1:**
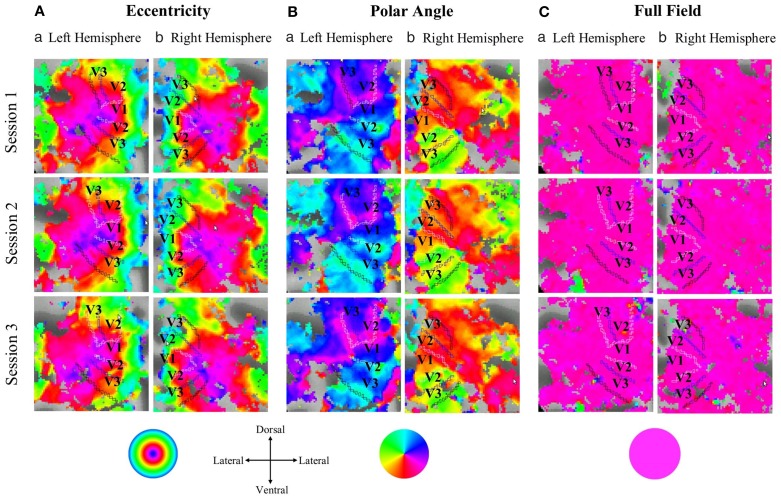
**Projection of the response phases onto the flattened representation of the occipital pole for the left and right hemisphere of a single representative subject during (A) eccentricity mapping, (B) polar angle mapping, and (C) full field stimulation (response threshold: ***p*** = 0.05)**. Typical eccentricity and polar angle maps were evident that covered the cortical expand activated during full field stimulation. There is a high degree of correspondence for the topographies across the three sessions within each stimulation condition.

**Figure 2:**
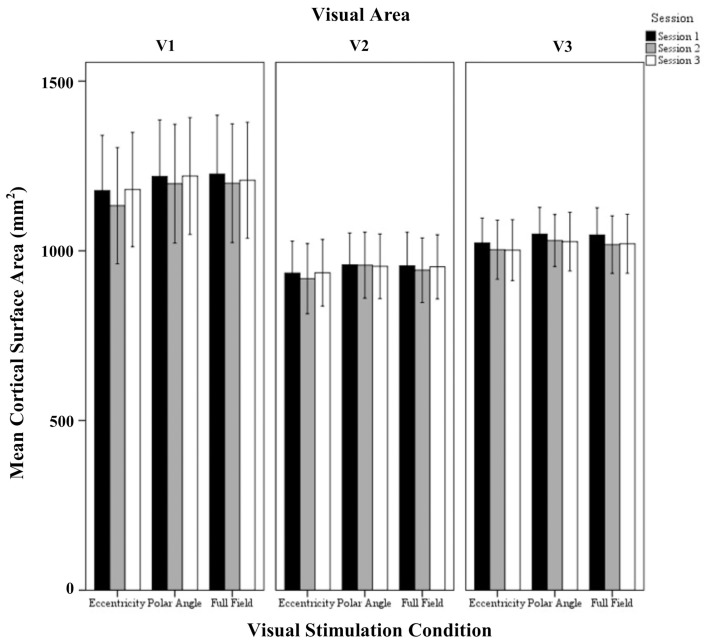
**Quantitative comparison of activated cortical surface area (mean ± SEM) across sessions in V1, V2, and V3 for all visual stimulation conditions**. Significant effects were observed for the factors session and visual area as detailed in Results.

**Figure 3:**
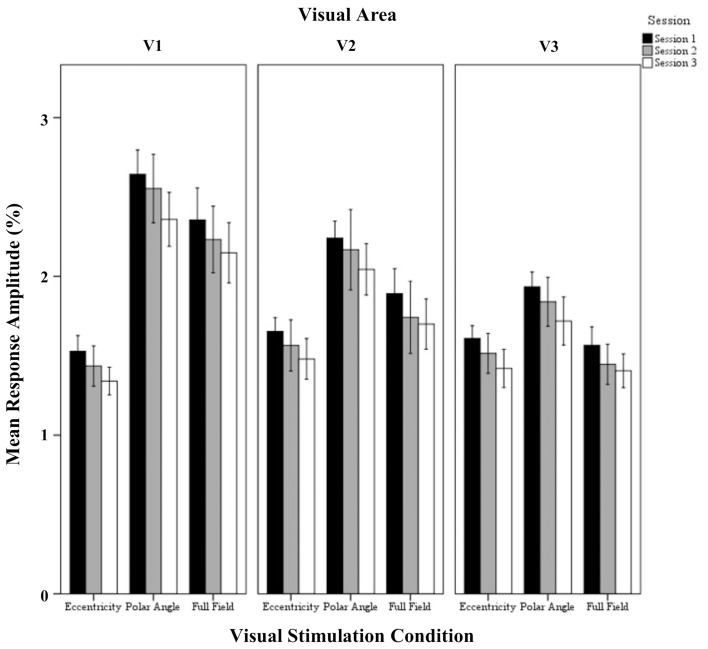
**Quantitative comparison of response amplitude (mean ± SEM) across sessions in V1, V2, and V3 for all visual stimulation conditions**. Significant effects were observed for the factors session, stimulation condition, and visual area, and for the interaction of visual stimulation condition and visual area as detailed in Results.

**Figure 4:**
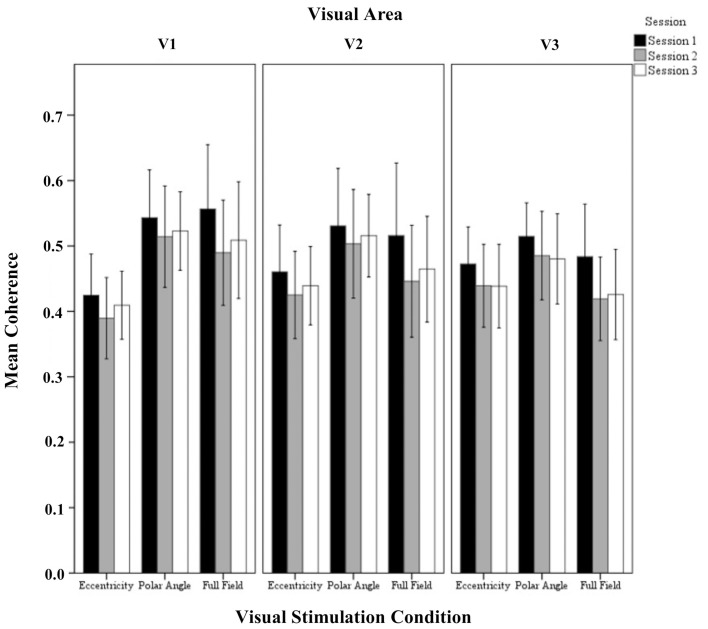
**Quantitative comparison of coherence (re-transformed mean ± SEM of Z-transformed value) across sessions in V1, V2, and V3 for all visual stimulation conditions**. Significant effects were observed for the factors session and visual stimulation condition as detailed in Results.

In Figure 1 flat maps of the occipital pole are depicted for one representative individual to indicate the typical response topography obtained for eccentricity mapping, polar angle mapping, and full field stimulation [see Supplementary information (Figure S5), for other subjects]. A high degree of consistency of the obtained response patterns across sessions 1–3 is evident. For a quantitative account of the effects we determined for each visual area the activated cortical surface area, i.e., the voxels with significant fMRI-responses (*p* < 0.05; Figure 2). Subsequently we determined for these super-threshold regions the average amplitude and coherence separately for each visual area and session (Figures 3, 4). The significance of the effects was determined with separate Three-Way repeated measures ANOVAs [factors: session, stimulation condition, visual area; see Table 1 and Supplementary information (Figures S6–S8)]. Initial Three-Way repeated measures ANOVAs comparing the dorsal and ventral parts of the early visual areas did not indicate any significant effect (individual and interaction factors) on the activated cortical surface area, response amplitude, and coherence (*p* > 0.05). Therefore, the dorsal and ventral parts of the early visual areas V1, V2, and V3 were combined for further analysis.

**Table 1:** **Results from repeated measures analyses for (a) activated cortical surface area, (b) responses amplitude, and (c) coherence**.

**Factor**	**(a) Activated cortical surface area**	**(b) Amplitude**	**(c) Coherence**
	*F*-ratio (df1, df2), *p*-value	*F*-ratio (df1, df2), *p*-value	*F*-ratio (df1, df2), *p*-value
Session	*F*_(2, 108)_ = 5.590, *p* = 0.005^*^	*F*_(2, 108)_ = 10.184, *p* < 0.001^*^	^a^*F*_(1.785,96.417)_ = 19.607, *p* < 0.001^*^
Visual stimulation condition	*F*_(2, 54)_ = 0.068, *p* = 0.934	*F*_(2, 54)_ = 18.467, *p* < 0.001^*^	*F*_(2, 54)_ = 7.242, *p* = 0.002^*^
Visual area	*F*_(2, 54)_ = 3.272, *p* = 0.046^*^	*F*_(2, 54)_ = 8.933, *p* < 0.001^*^	*F*_(2, 54)_ = 0.673, *p* = 0.514
Session × Visual area	*F*_(4, 108)_ = 1.198, *p* = 0.316	*F*_(4, 108)_ = 0.057, *p* = 0.994	^a^*F*_(3.571, 96.417)_ = 0.2666, *p* = 0.881
Session × Visual stimulation condition	*F*_(4, 108)_ = 0.405, *p* = 0.804	*F*_(4, 108)_ = 0.192, *p* = 0.942	^a^*F*_(3.571, 96.417)_ = 1.609, *p* = 0.184
Visual stimulation condition × Visual area	*F*_(4, 54)_ = 0.005, *p* = 1.000	*F*_(4, 54)_ = 3.247, *p* = 0.019^*^	*F*_(4, 54)_ = 1.381, *p* = 0.253
Session × Visual stimulation condition × Visual area	*F*_(8, 108)_ = 0.210, *p* = 0.989	*F*_(8, 108)_ = 0.023, *p* = 1.000	^a^*F*_(7.142, 96.417)_ = 0.027, *p* = 1.000

a *Greenhouse-Geisser correction*.

* *The effect factor is significant at the 0.05 level (p < 0.05)*.

#### Activated cortical surface area

The repeated measures ANOVA indicated significant effects of the factors session (*p* = 0.005) and visual area (*p* = 0.046), but not visual stimulation condition. No significant interactions of the factors were evident. *Post-hoc* tests were performed to detail the effect of session and visual areas. Importantly, for the factor session, there was a small, but significant reduction of activated area from sessions 1 to 2 (1066 ± 40 mm^2^ vs. 1044 ± 42 mm^2^; *p* = 0.006). Furthermore, for the factor visual areas, V1 exceeded V2 significantly (1196 ± 71 mm^2^ vs. 946 ± 71 mm^2^; *p* = 0.045) which is in accordance with Dougherty et al. (2003).

#### Response amplitude

The repeated measures ANOVA indicated highly significant effects of the factors session (*p* < 0.001), visual stimulation condition (*p* < 0.001), and visual area (*p* < 0.001) on response amplitude and a significant interaction only for visual stimulation condition × visual area (*p* = 0.019). Importantly, *post-hoc* tests showed a significant amplitude reduction from sessions 1 to 3 (1.94 ± 0.04% vs. 1.74 ± 0.05%; *p* < 0.001). Furthermore, for the factor visual stimulation, *post-hoc* tests showed increased amplitudes for (1) polar angle compared to eccentricity (2.17 ± 0.08% vs. 1.51 ± 0.08%; *p* < 0.001), (2) full field compared to eccentricity (1.83 ± 0.08% vs. 1.51 ± 0.08%; *p* = 0.012), and (3) polar angle compared to full field (2.17 ± 0.08% vs. 1.83 ± 0.08%; *p* = 0.010). Significant differences for the response amplitudes of visual stimulation conditions were expected as they have different stimulation duty cycles, which influences the Fourier-analysis-based quantification of the response amplitudes and coherences (see Materials and Methods). For the factor visual area *post-hoc* tests showed greater amplitudes in V1 than V3 (2.07 ± 0.08% vs. 1.61 ± 0.08%; *p* < 0.001), which is in accordance with Hoffmann et al. (2009).

#### Coherence

The repeated measures ANOVA indicated significant differences for session and visual stimulation condition. No significant interactions of the factors were evident. Importantly, for the factor session, *post-hoc* tests showed a significant reduction from (1) sessions 1 to 2 (0.55 ± 0.01 vs. 0.50 ± 0.01 *p* < 0.001) and (2) sessions 1 to 3(0.55 ± 0.01 vs. 0.51 ± 0.01 *p* < 0.001). Furthermore, for the factor visual stimulation, *post-hoc* tests showed that coherences were greater for polar angle compared to eccentricity (0.57 ± 0.02 vs. 0.46 ± 0.02; *p* = 0.001). Similar to the effect of stimulation on the amplitude of the BOLD signal, it is assumed that this is due to the effects of the different stimulation duty cycles for the different conditions on the Fourier-based analysis of the BOLD signals (see Materials and Methods and above).

Taken together, effects of the factor session, i.e., a reduction of the measures in the later sessions compared to session 1, were evident for each measure tested, independent of the visual area analyzed. Specifically, activated cortical surface area decreased from sessions 1 to 2 by 2% (*p* < 0.006), response amplitudes decreased from sessions 1 to 3 by 11.5% (*p* < 0.001), and coherence decreased from sessions 1 to 2 by 10% (*p* < 0.001) and session 3 by 8% (*p* < 0.001). Importantly, no session effects between sessions 2 and 3 were evident. This suggests that in session 1 a “novelty effect” contributed to the obtained measures as discussed below. From the scatter of the data, specifically the 95% confidence interval of session 2, the minimal effect sizes can be inferred that can be detected with a 5% significance threshold (*p* < 0.05) for a design similar to that of the present study, i.e., 7 subjects, repeated measures design, and an initial scan to eliminate novelty effects. The 95% confidence interval ranges for cortical surface area ±1.5% of the mean cortical surface area, for response amplitude ±6% for the mean percentage of the amplitude modulation, and for coherence ±5% of the Z-transformed coherence.

### Reproducibility of phase maps

**Figure 5:**
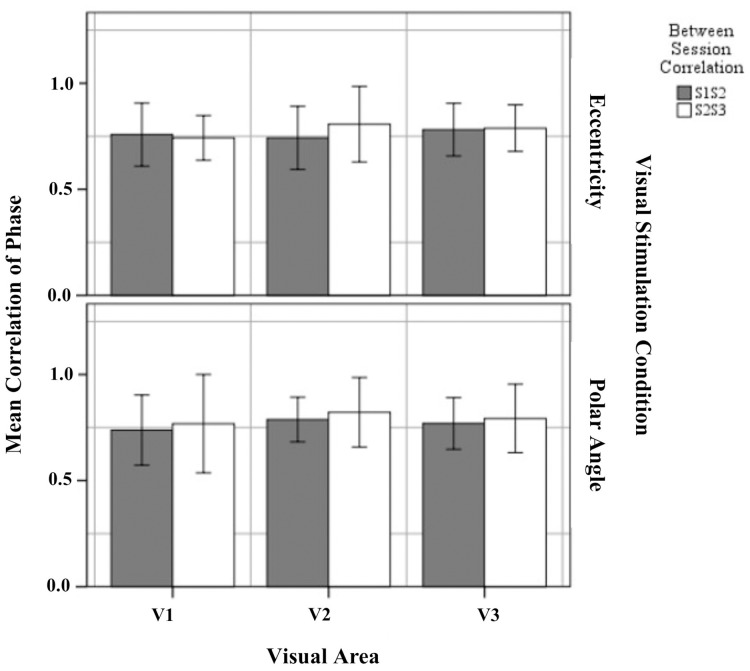
**Quantitative comparison of correlation of phase maps (re-transformed mean ± SEM of Z-transformed value) of between sessions 1 and 2, and between sessions 2 and 3 within defined visual for eccentricity and polar angle**. No significant effects were observed as detailed in Results.

The phase map similarity or reproducibility within defined visual areas for eccentricity and polar angle were determined (1) between sessions 1 and 2 (S1S2) and (2) between sessions 2 and 3 (S2S3). We applied a threshold of *p* = 0.05 for all visual areas in both sessions for these analyses. Figure 5 shows the mean correlation coefficient of S1S2 and S2S3 within the visual areas for eccentricity and polar angle. The results showed that phase maps in V1, V2, and V3, for both eccentricity and polar angle, were highly correlated, with a mean correlation coefficient >0.70. This is in agreement with a previous studies on intra-session variability (Hoffmann et al., 2012) and exceeds the value of a study on inter-session variability (Swisher et al., 2007).

Next, the significance of the variability on the Z-transform correlation between session group (S1S2 and S2S3) was determined separately for each visual area (V1, V2, and V3) and visual stimulation (eccentricity and polar angle). The results showed that the difference between the mean correlation coefficient in S1S2 and S2S3 was not statistically significant for eccentricity mapping within visual area V1 [*t*_(12)_ = 0.388, *p* = 0.705], V2 [*t*_(12)_ = −1.404, *p* = 0.186], and V3 [*t*_(12)_ = −0.211, *p* = 0.386] and for polar angle mapping within visual areas V1 [*t*_(12)_ = −0.484, *p* = 0.637], V2 [*t*_(12)_ = −0.938, *p* = 0.345], and V3 [*t*_(12)_ = −0.600, *p* = 0.560]. It is concluded that the phase maps for both eccentricity and polar angle mapping correlate highly across sessions and that there is no systematic intersession effect on the obtained mapping.

### Head motion

**Figure 6:**
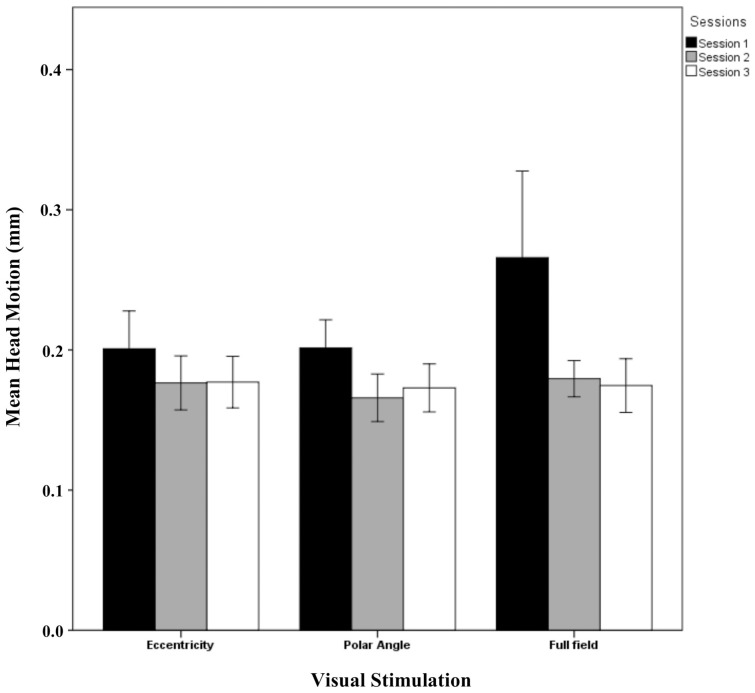
**Quantitative comparison (mean ± SEM) of head movement across sessions for all visual stimulation conditions**. No significant effects were observed as detailed in Results.

The results showed that the phase maps were stable across sessions. However, the activation between sessions indicated significant differences from sessions 1 to 2 and 3 whereas no session effects from sessions 2 to 3 were evident. A potential cause of different BOLD responses in session 1 compared to the others might be a different extent of head motion in session 1. Figure 6 shows the mean head motion across sessions. Overall, the mean head motion was < 0.3 mm over time in all stimulation conditions. A repeated measures One-Way ANOVA (factors: session and stimulation condition; see Table 2) did not indicate any significant effects on head movement (*p* > 0.05), neither for the individual factors nor for their interaction. A non-significant trend for the factor session (*p* = 0.074), mainly a reduction of head movement by 0.049 mm from sessions 1 to 2, was observed. As the scatter was particularly high for the full field stimulation condition, we excluded this condition in a subsequent analysis. Again, a similar non-significant trend for the factor time (*p* = 0.160) was observed (a reduction of head movement by 0.030 mm). In conclusion, these results do not support head motion as a major cause of the observed novelty effect in the BOLD responses for session 1. This is further underlined by the individual response patterns given in Supplementary information (Additional results and Figures S1–S4).

**Table 2:** **Results from repeated measures analyses of the head movement for (a) eccentricity, polar angle and full field and (b) eccentricity and polar angle**.

**Factor**	**(a)**	**(b)**
	*F*-ratio (df1, df2), *p*-value	*F*-ratio (df1, df2), *p*-value
Session	^a^*F*_(1.185, 21.322)_ = 3.3777, *p* = 0.074	^a^*F*_(1.128, 13.537)_ = 0.204, *p* = 0.160
Visual stimulation condition	*F*_(2, 18)_ = 0.276, *p* = 0.762	*F*_(1, 12)_ = 0.024, *p* = 0.879
Sessions × Visual stimulation condition	^a^*F*_(2.369, 21.322)_ = 0.615, *p* = 0.576	^a^*F*_(1.128, 13.537)_ = 0.066, *p* = 0.830

a *Greenhouse-Geisser correction*.

## Discussion

We assessed the inter-session variability of fMRI, for retinotopic mapping and full field stimulation and report sizable, but reduced BOLD responses after the initial session as reflected by decreased activated cortical surface area from sessions 1 to 2, decreased response amplitude from sessions 1 to 3, and decreased coherence from sessions 1 to 2 and 3. Importantly, the findings show no session effect from sessions 2 to 3 and do not support head motion as a major cause of the higher BOLD responses in session 1. Additionally, we examined the correlation coefficient of phase maps between sessions 1 and 2, and between sessions 2 and 3. The results showed that phase maps in early visual areas were highly correlated for eccentricity and polar angle and the correlation of session 2 with sessions 1 and 3 did not differ significantly, demonstrating that the phase maps and the cortical representations were stable.

In the present study, the BOLD responses are reduced after the first session, which might be related to a “novelty effect” associated with this initial scan. This corresponds to several previous reports. Raemaekers et al. (2007) observed a reduction in activation from sessions 1 to 2 (1 week intervals between sessions) in an anti-and prosaccade study. The observed amplitude reduction may reflect lower subject alertness in the follow-up sessions due to familiarity with the procedure (Raemaekers et al., 2007). In a motor task with a stop signal paradigm Zandbelt et al. (2008) did not find inter-session effects in the BOLD signal changes but they observed a reduction of cortical surface area from sessions 1 to 2 (1 week intervals between sessions) and no session effect from sessions 2 to 3 (1 week intervals between sessions). Another potential neuronal mechanism proposed for response decreases is increased neural efficiency with experience (Poldrack, 2000; Kelly et al., 2006; Zandbelt et al., 2008). It is suggested that fewer neurons are needed to fire strongly in response to a particular task (Poldrack, 2000) or a more precise functional circuit is recruited (Garavan et al., 2000). An alternative cause for a reduction in the brain activation pattern might be associated with inter-session differences in head motion during the MRI scans. Head movement can lead to erroneous conclusions in fMRI even for normal subjects (Savoy, 2005). In our analysis, however, there was an absence of session-dependence of the head movements, which favors the “novelty effect” hypothesis. It should be noted that several sources of noise influence fMRI data, i.e., physiological (e.g., respiratory, cardiovascular, motion artifacts) and technical noise (e.g., differences in viewing distance, susceptibility). While these noise intrusions enhance the variance of the obtained fMRI data, they are not expected to introduce systematic inter-session effects as observed in the present study. Such noise effects are independent of the session sequence, as has here been tested explicitly for head motion.

The presented data suggest that longitudinal studies on visually driven cortical activity can be confounded by sequential effects, particularly in the initial scan. This is of particular relevance in the face of current therapeutic initiatives to improve or even restore visual function in patients. Visually driven fMRI responses appear to be powerful biomarkers for the efficacy of these treatments (e.g., Ashtari et al., 2011, 2014; Baseler et al., 2011). The present study underscores that a simple baseline vs. post-treatment comparison of fMRI responses may be error-prone. Any future longitudinal fMRI study should therefore carefully evaluate whether the applied task induces stable activation quantified by the respective outcome measure of the study. Only if this is established can all time points be included in the evaluation. In this study we demonstrate, for the visual stimulation paradigm applied, that the initial MRI session may not be representative and could lead to erroneous conclusions in longitudinal fMRI studies. It appears that this might be resolved by the inclusion of a control group, which will show sequential effects that resemble those seen in the patients. It should be noted, however, that in this case, the patient and control group, in addition to the standard age- and sex-matched, should also be matched according to their experience with the scanning paradigm. Furthermore, it is not clear whether novelty effects scale with visual impairment. Therefore, an alternative approach should be taken into consideration. Implementation of initial scanning sessions can contribute to the stability of the BOLD-fMRI signal. This is possible because no inter-session effects between sessions 2 and 3 were evident in this study, both in cortical surface area, coherence, and also in amplitude, even though we can see a linear drop of amplitude (Figure 3) from sessions 1 to 3. This absence of significant differences in the mean amplitude between sessions 2 and 3 does not proof the equality of the data, however, it does not show relevant response instabilities either. Importantly, this effect may last for several months, which was the inter-session interval that we used. Consequently, an initial MRI session (indicated as session 1 in this study) before a baseline scan (indicated as session 2 in this study) should be considered in longitudinal study designs. In practice, this initial session could be used to acquire additional MRI scans that are required only once (e.g., anatomical scans, functional localisers). This way, all scans entering the actual longitudinal observation can follow the exact same sequence and protocol. Our experimental design was to scan a sample of 7 subjects at 7 T, leading to a sensitivity that allowed determination of inter-session changes of cortical surface area, response amplitude, and coherence, at 1.5, 6, and 5%, respectively.

Taken together, reduced cortical surface areas, amplitudes, and coherences of visually driven BOLD responses were found for follow-up sessions in comparison to an initial scanning session, while the phase maps were reproducible across sessions. Cognitive effects such as novelty, learning, and adaptation are plausible reasons for the observed inter-session effects. As a consequence, data of an initial session in a longitudinal study might not be representative. This should be taken into consideration when interpreting fMRI results from longitudinal studies.

## Author contributions

Substantial contributions to the conception or design of the work, and/or acquisition of the data, and/or analysis and interpretation of the data: AH, OS, MH; Drafting the work or revising it critically for important intellectual content: AH, OS, MH; Final approval of the version to be published: OS, MH; Agreement to be accountable for all aspects of the work in ensuring that questions related to the accuracy or integrity of any part of the work are appropriately investigated and resolved: AH, OS, and MH.

### Conflict of interest statement

The authors declare that the research was conducted in the absence of any commercial or financial relationships that could be construed as a potential conflict of interest.
