# Supplementary material for: Quantitative assessment of visual cortex function with fMRI at 7 Tesla—test–retest variability
Source: Front Hum Neurosci. 2015 Sep 1;9:477. doi: 10.3389/fnhum.2015.00477 (PMC4555013; doi:10.3389/fnhum.2015.00477)
Supplement: Supplementary file 1 [file DataSheet1.DOCX]

**Supporting Information S1:** Additional results and supplemental figures.

**Additional Results**

Figures S1-S3 show the individual subject patterns over session for cortical surface area, response amplitude and coherence. Except for subjects 1 and 7, all subjects had undergone fMRI scans previously; only one of these, subject 6, was experienced in retinotopic mapping. While inexperienced subject 7 largely followed the global trends, inexperienced subject 1 generally showed a slight increase in response amplitude and coherence. The pattern differences might be due to head movements in subject 1, since greater head movement was detected as compared to the other subjects, except subject 3. Experienced subject 3 also showed comparatively large head movement values (Fig. S4). Yet these two subjects were not excluded from this study because the head movement values were clearly less than 1 mm leaving the data uncorrupted by our definition. In addition, the head movement for subject 3 only showed a different pattern for the first session for eccentricity and full field stimulation. In conclusion, in the present study the difference in head motion between experienced and inexperienced subjects appears to be of marginal importance.

**Supplemental Figures** (separate page below)

**Fig S1.** The activated cortical surface area (mm^2^) across sessions in V1, V2 and V3 for all visual stimulation conditions for subjects 1-7.

**Fig. S2.** The response amplitude (%) across sessions in V1, V2 and V3 for all visual stimulation conditions for subjects 1-7.

**Fig. S3.** The coherence (Z-transformed values) across sessions in V1, V2 and V3 for all visual stimulation conditions for subjects 1-7.

**Fig. S4.** The head motion (mm) across sessions for all visual stimulation conditions for subjects 1-7.

**Fig. S5.** Projection of the response phases onto the flattened representation of the occipital pole for the left and right hemisphere for (a) subject 1, (b) subject 2, (c) subject 3, (d) subject 4, (e) subject 5, (f) subject 6, and (g) subject 7, during eccentricity mapping, polar angle mapping and full field stimulation (response threshold: *p*=0.05). Typical eccentricity and polar angle maps were evident that covered the cortical expand activated during full field stimulation.

**Fig. S6.** Quantitative comparison of cortical surface area (mean ± SEM) across sessions in V1, V2 and V3 for all visual stimulation conditions. * *p*<0.05.

**Fig. S7.** Quantitative comparison of response amplitude (mean ± SEM) across sessions in V1, V2 and V3 for all visual stimulation conditions. * *p*<0.05.

**Fig. S8.** Quantitative comparison of coherence (re-transformed mean ± SEM of Z-transformed value) across sessions in V1, V2 and V3 for all visual stimulation conditions. * *p*<0.05.


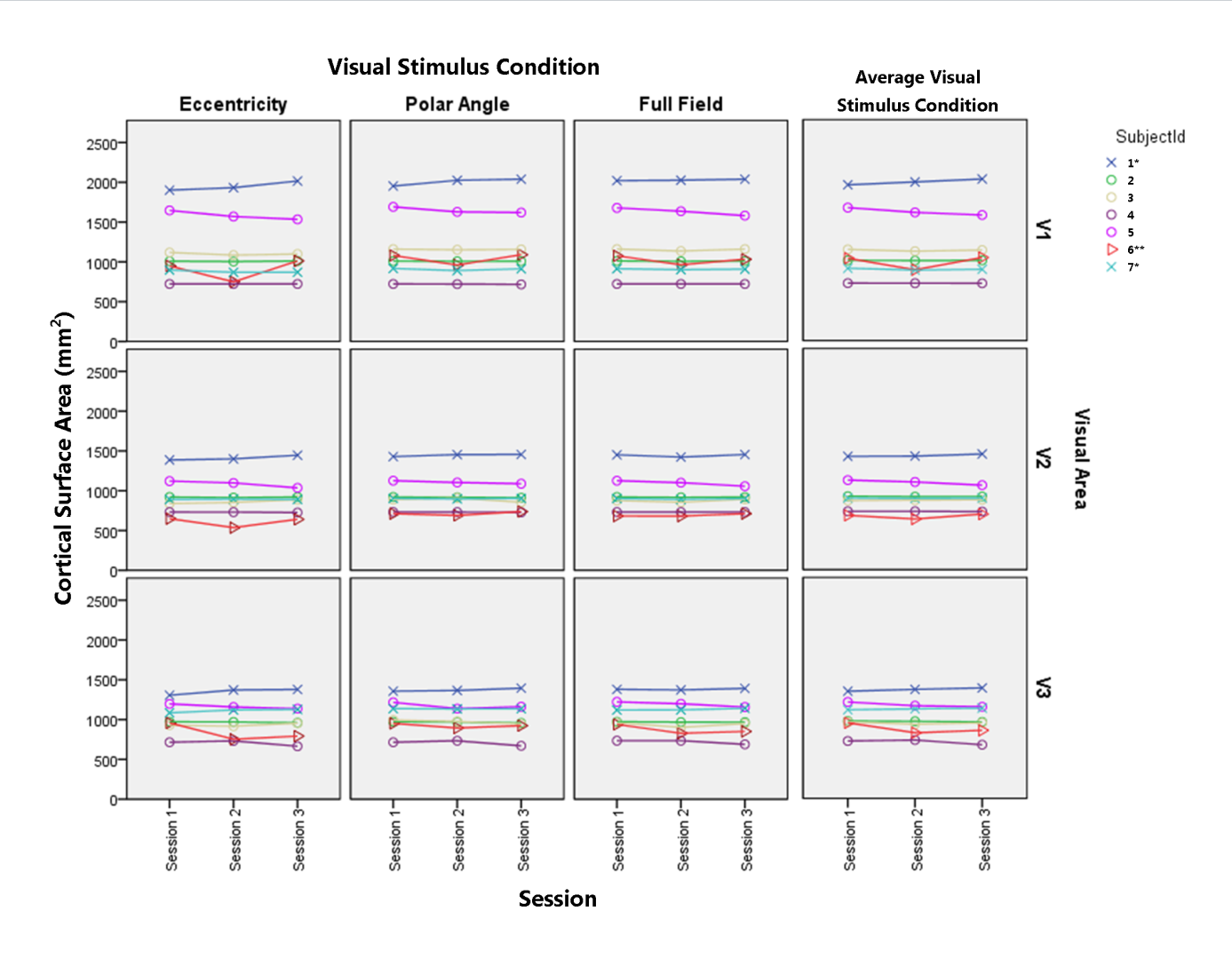


Fig. S1. The activated cortical surface area (mm^2^) across sessions in V1, V2 and V3 for all visual stimulation conditions for subjects 1-7.

Note: * Inexperienced subject; ** Experienced subject in retinotopic experiment (in different study)


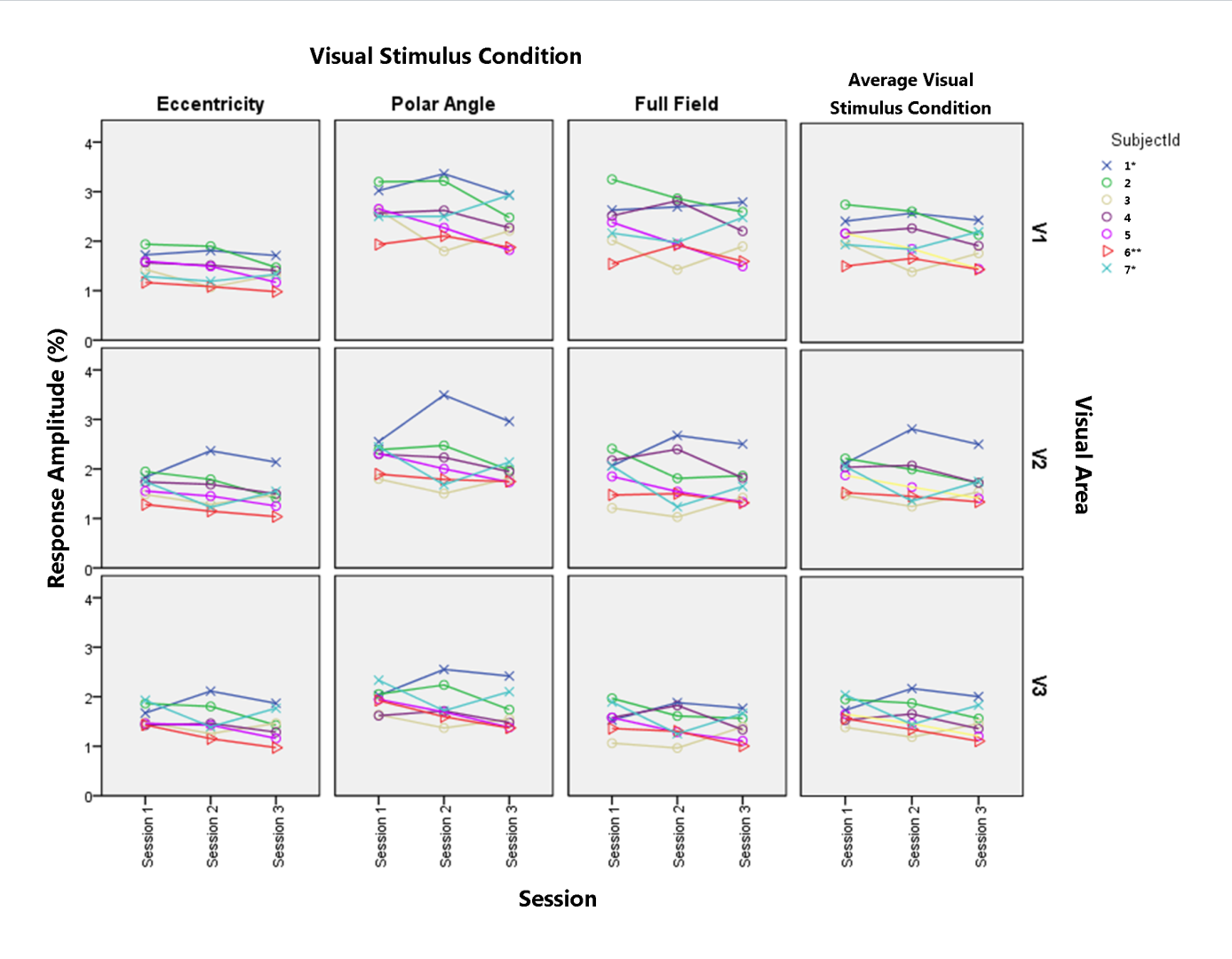


Fig. S2. The response amplitude (%) across sessions in V1, V2 and V3 for all visual stimulation conditions for subjects 1-7.

Note: * Inexperienced subject; ** Experienced subject in retinotopic experiment (in different study)


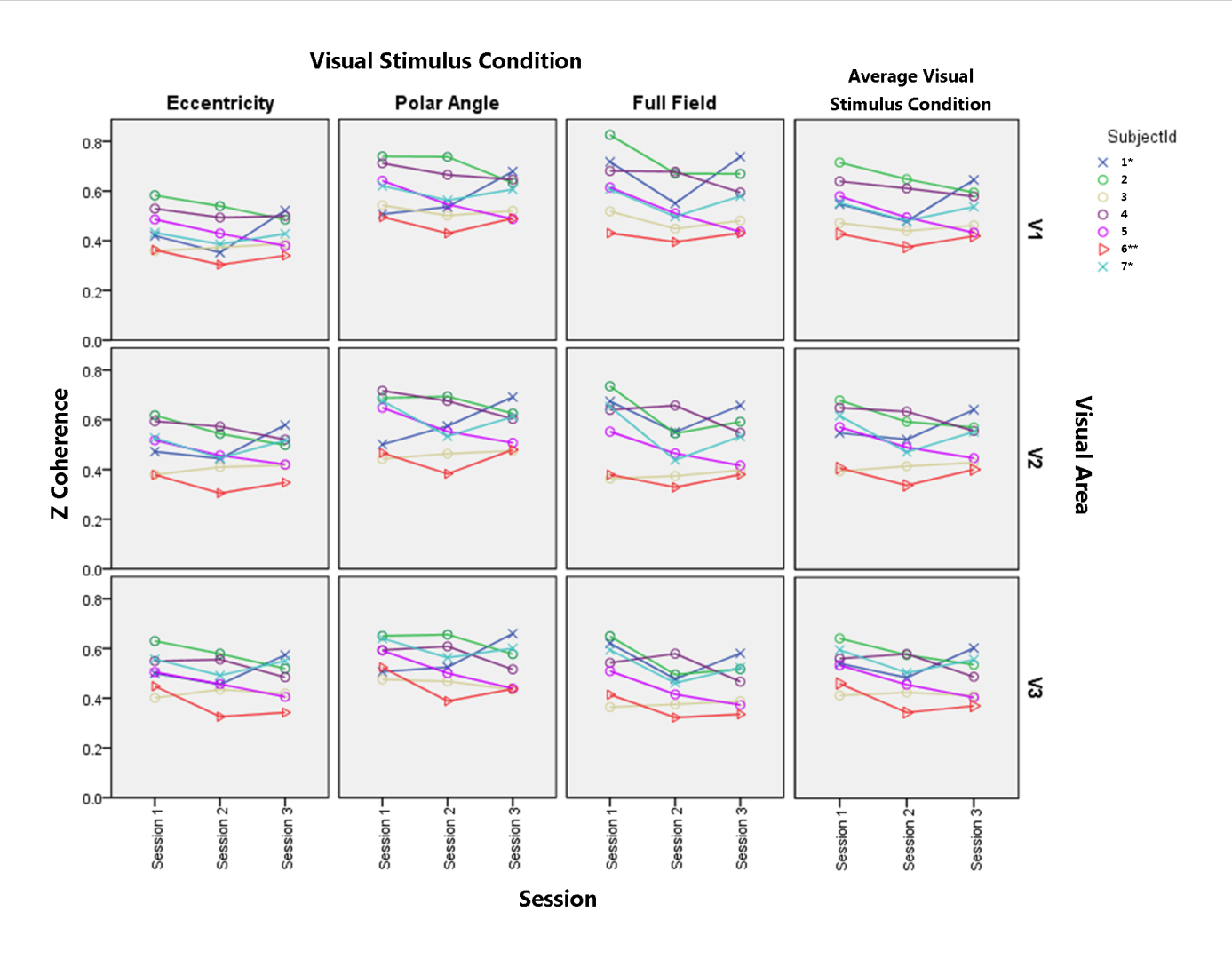


Fig. S3. The coherence (Z-transformed values) across sessions in V1, V2 and V3 for all visual stimulation conditions for subjects 1-7.

Note: * Inexperienced subject; ** Experienced subject in retinotopic experiment (in different study)

**
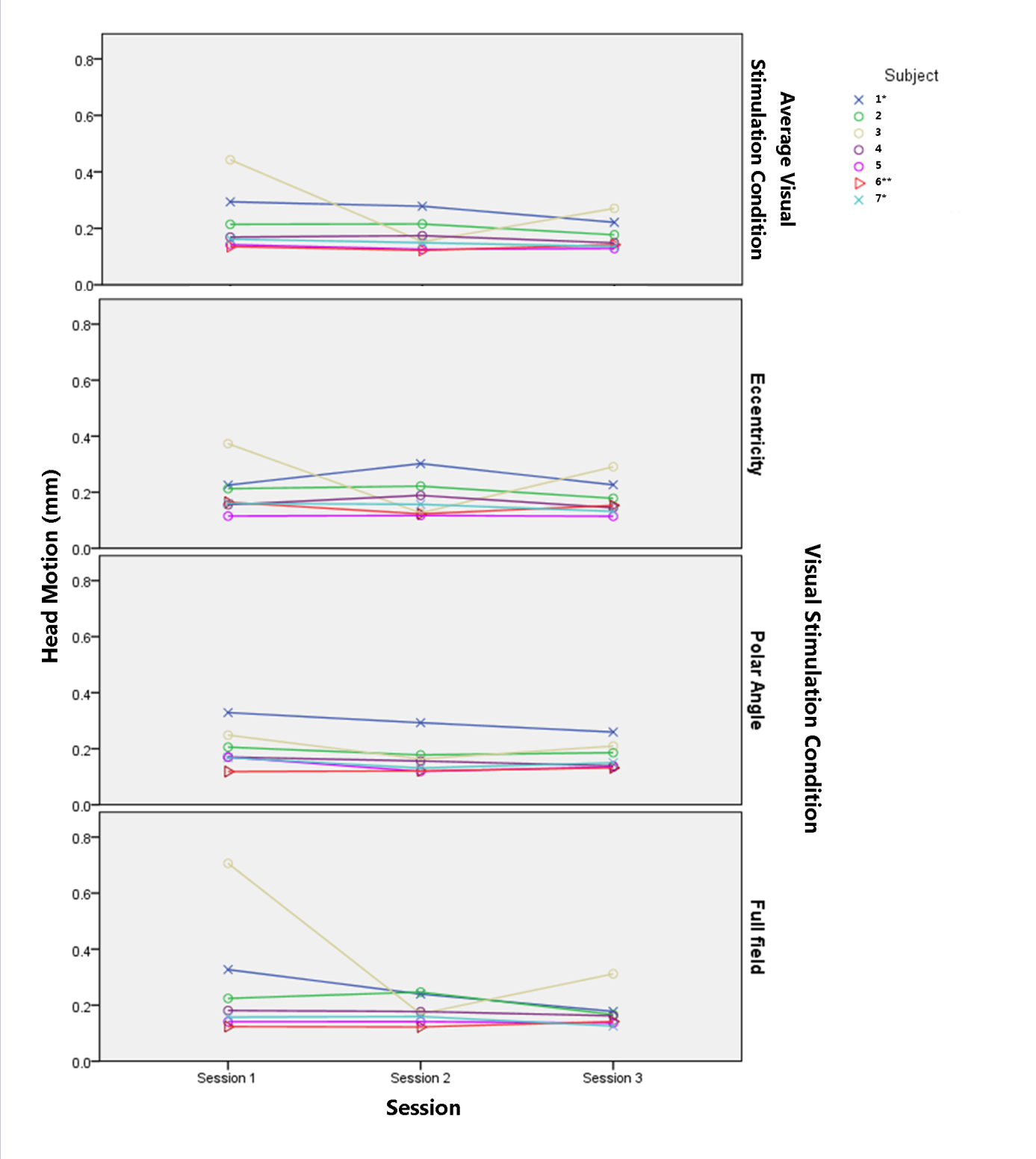
**

Fig. S4. The head motion (mm) across sessions for all visual stimulation conditions for subjects 1-7.

Note: * Inexperienced subject; ** Experienced subject in retinotopic experiment (different study)


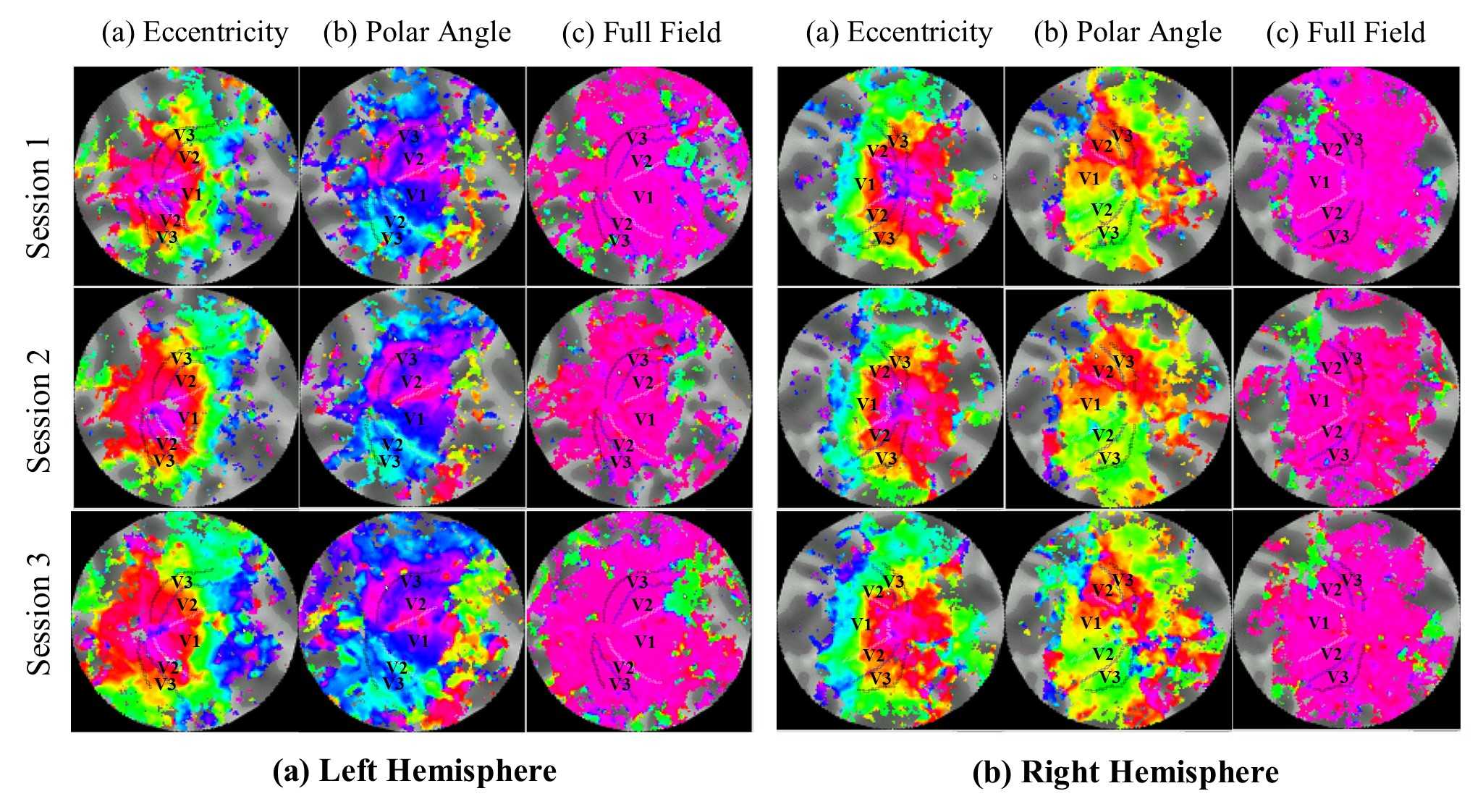


1. subject 1


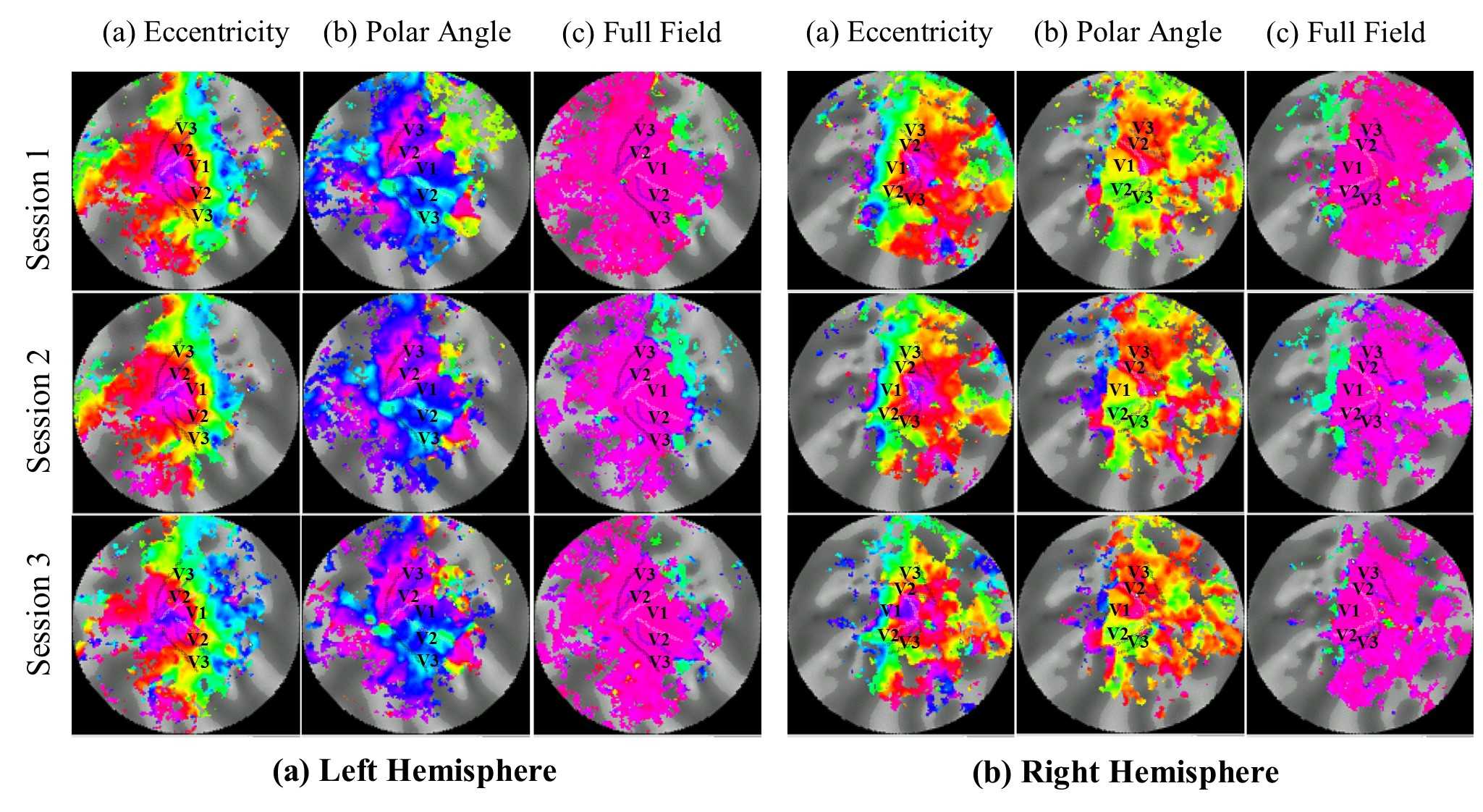


1. subject 2


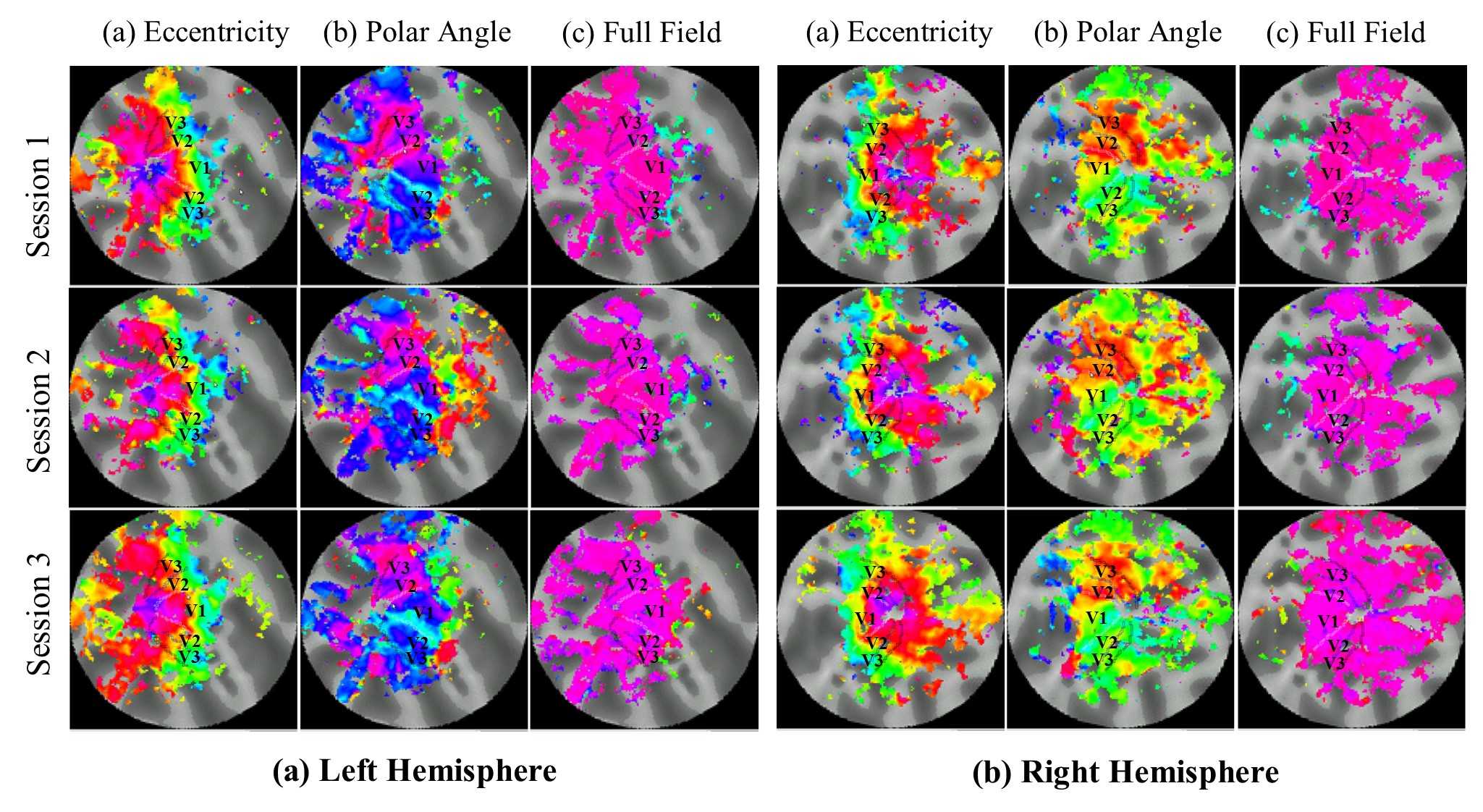


1. subject 3


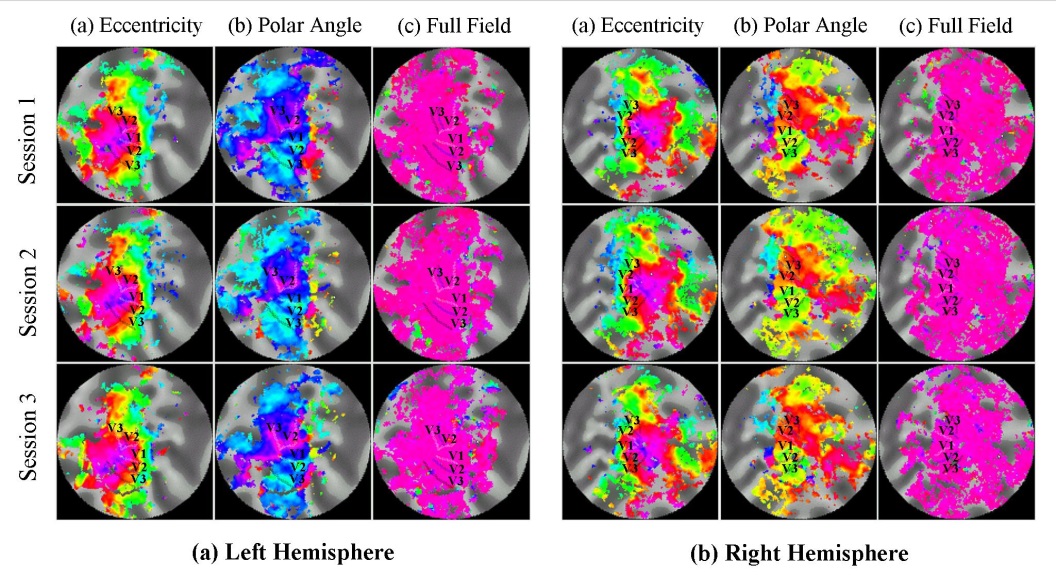


1. subject 4


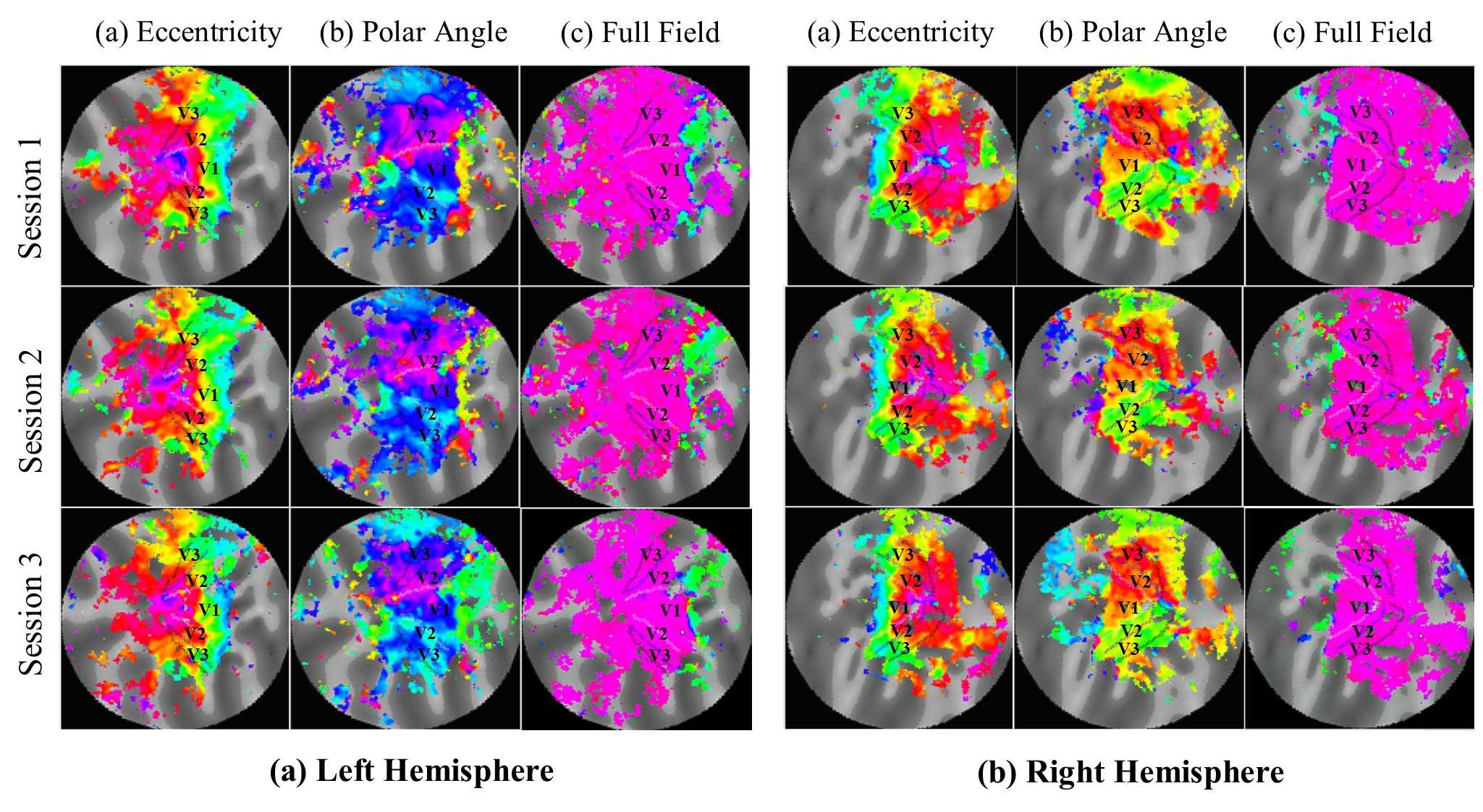


1. subject 5


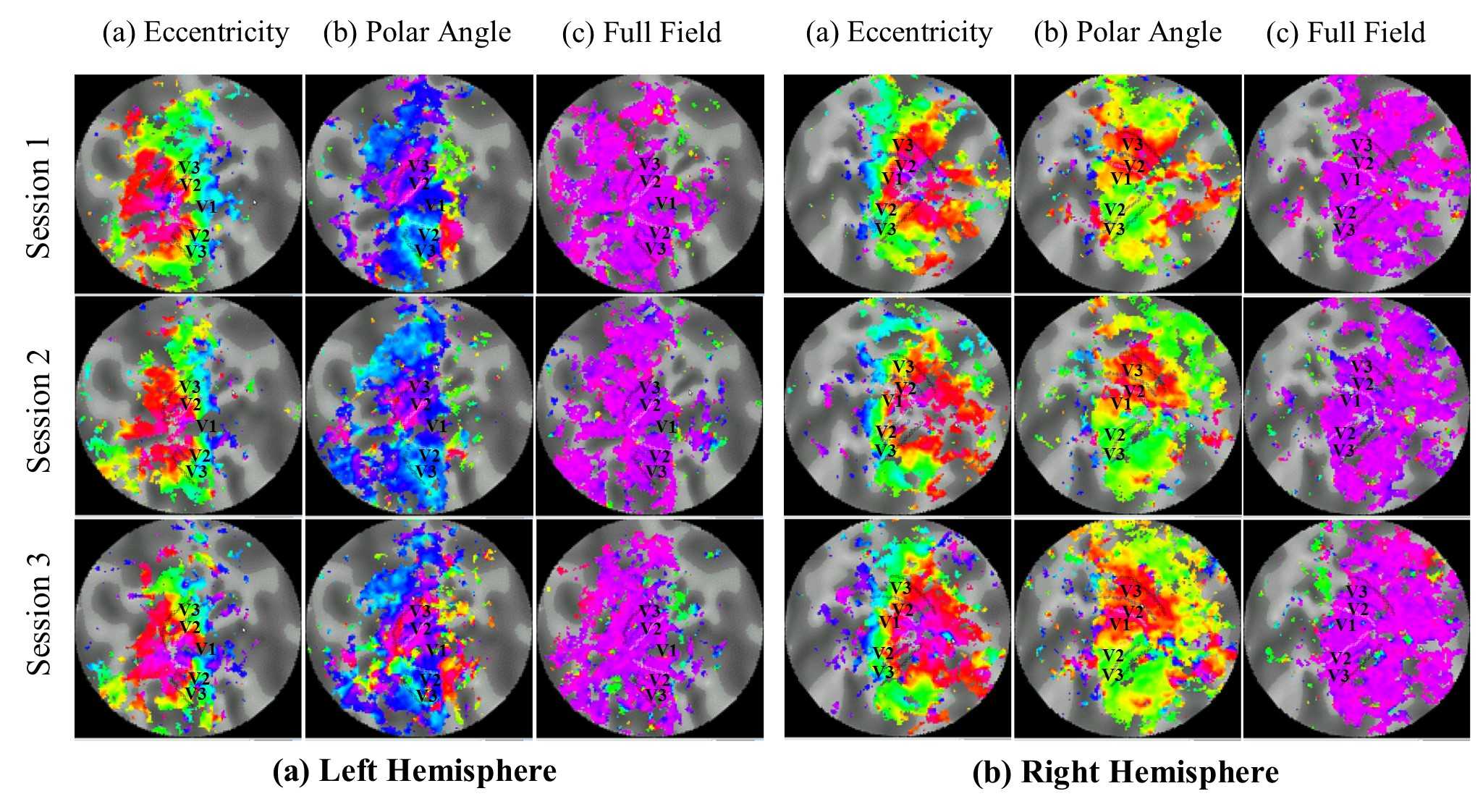


1. subject 6


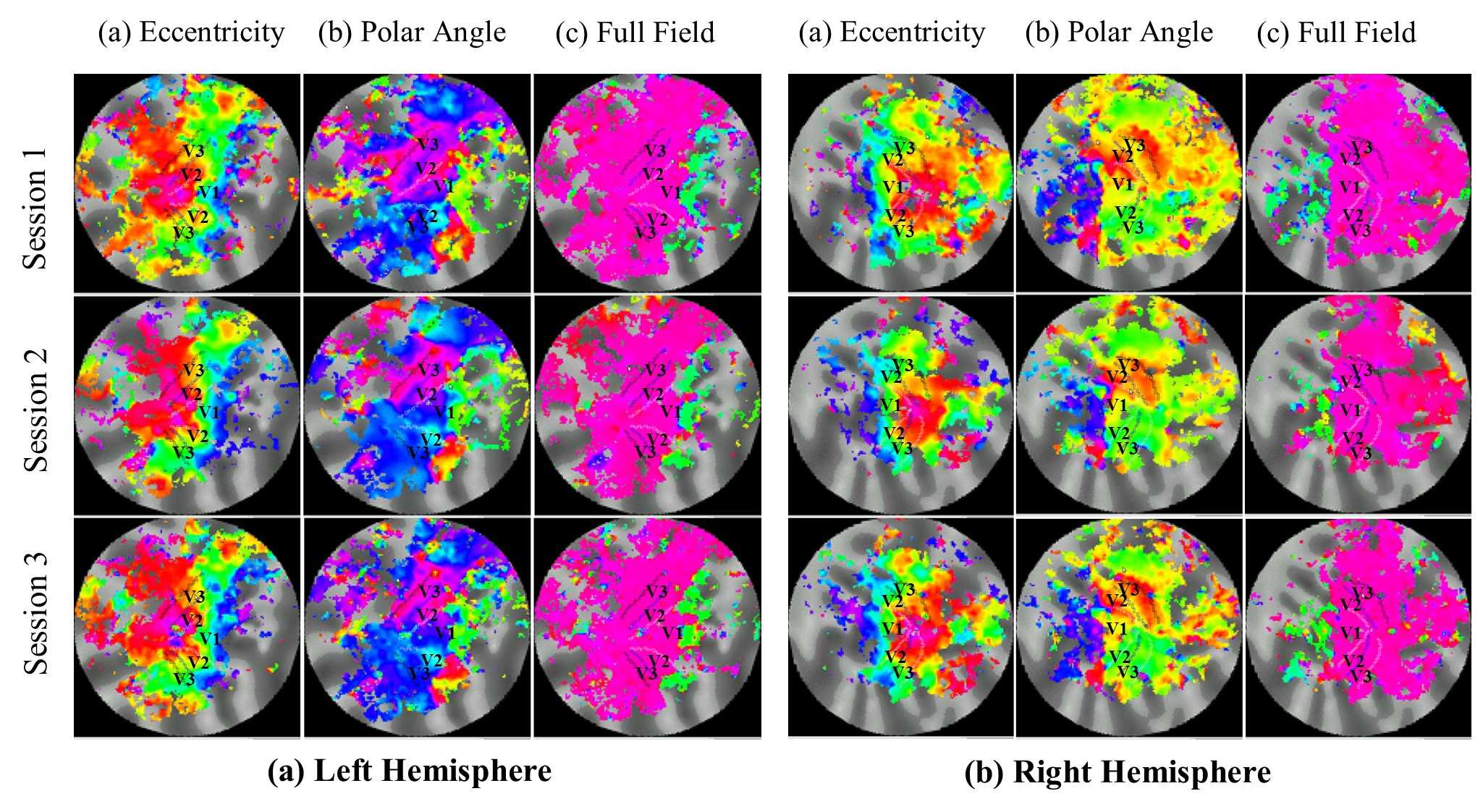


1. Subject 7

Fig. S5. Projection of the response phases onto the flattened representation of the occipital pole for the left and right hemisphere for (a) subject 1, (b) subject 2, (c) subject 3, (d) subject 4, (e) subject 5, (f) subject 6, and (g) subject 7, during eccentricity mapping, polar angle mapping and full field stimulation (response threshold: *p*=0.05). Typical eccentricity and polar angle maps were evident that covered the cortical expanse activated during full field stimulation.


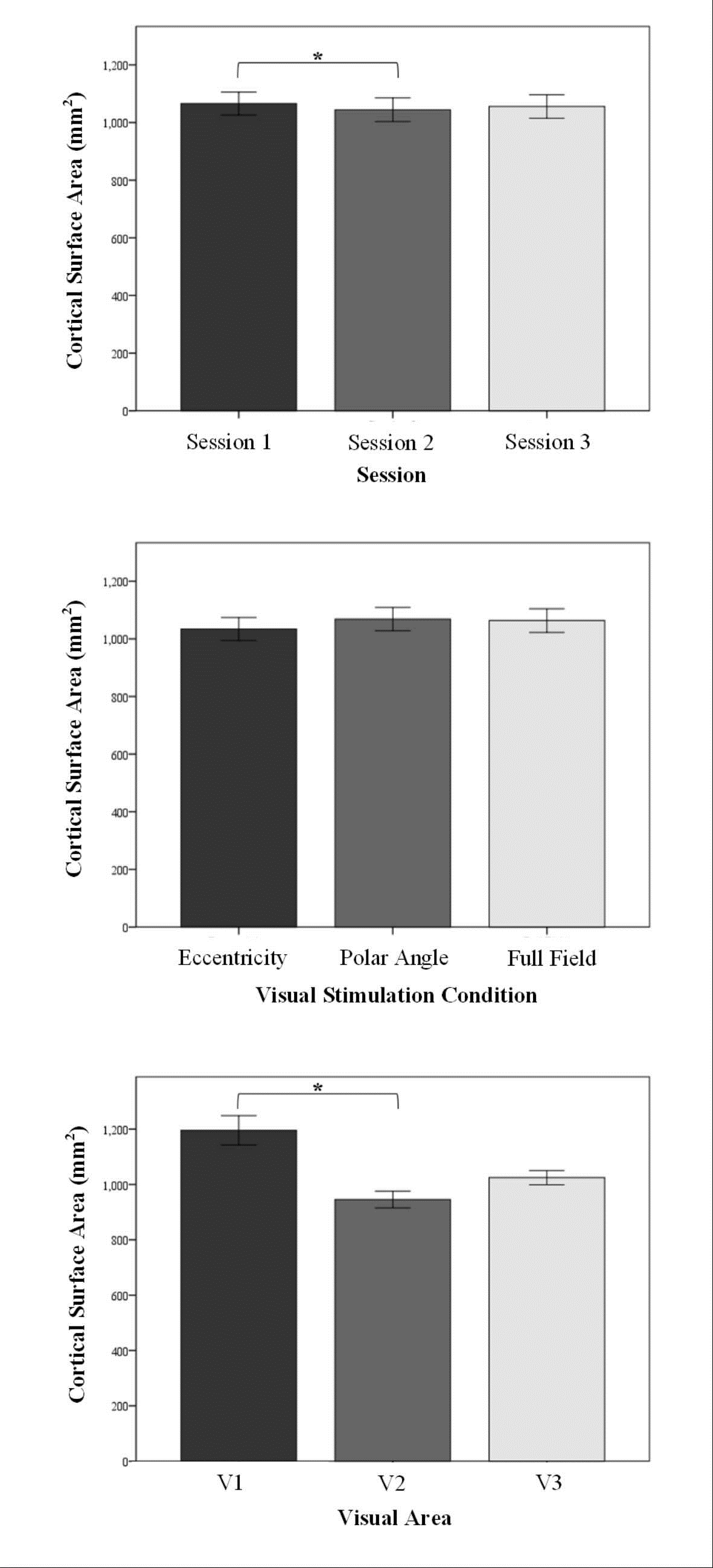


Fig. S6. Quantitative comparison of cortical surface areas (mean ± SEM) across sessions in V1, V2 and V3 for all visual stimulation conditions. **p*<0.05.


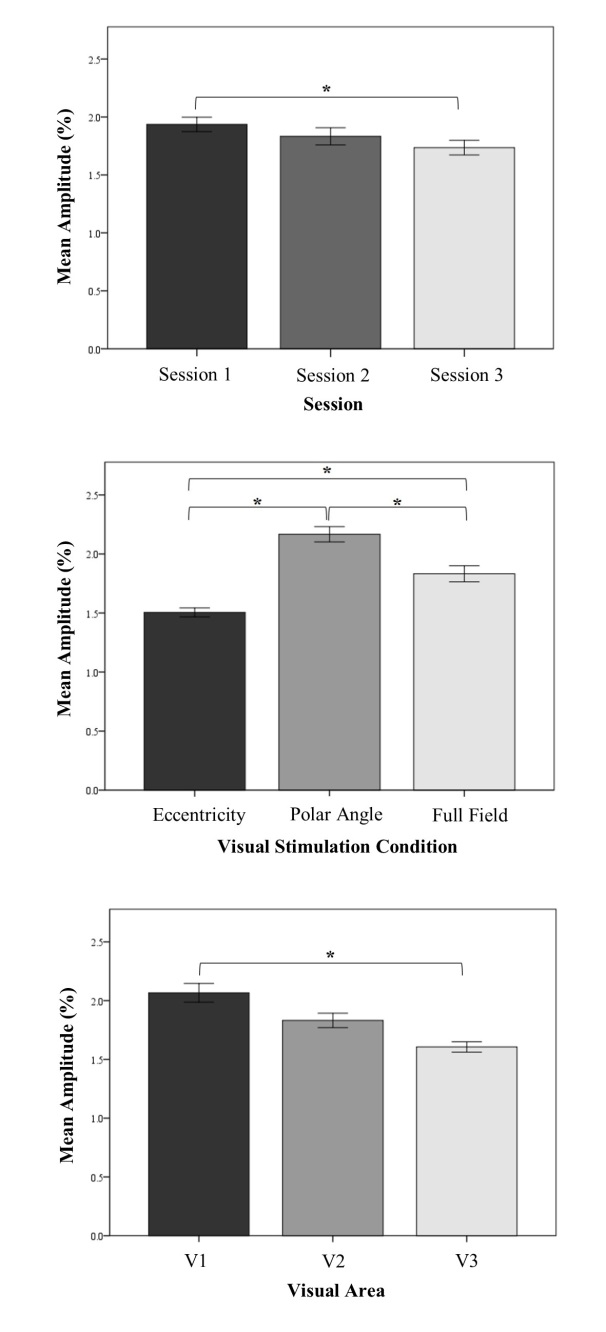


Fig. S7. Quantitative comparison of response amplitude (mean ± SEM) across sessions in V1, V2 and V3 for all visual stimulation conditions. **p*<0.05.


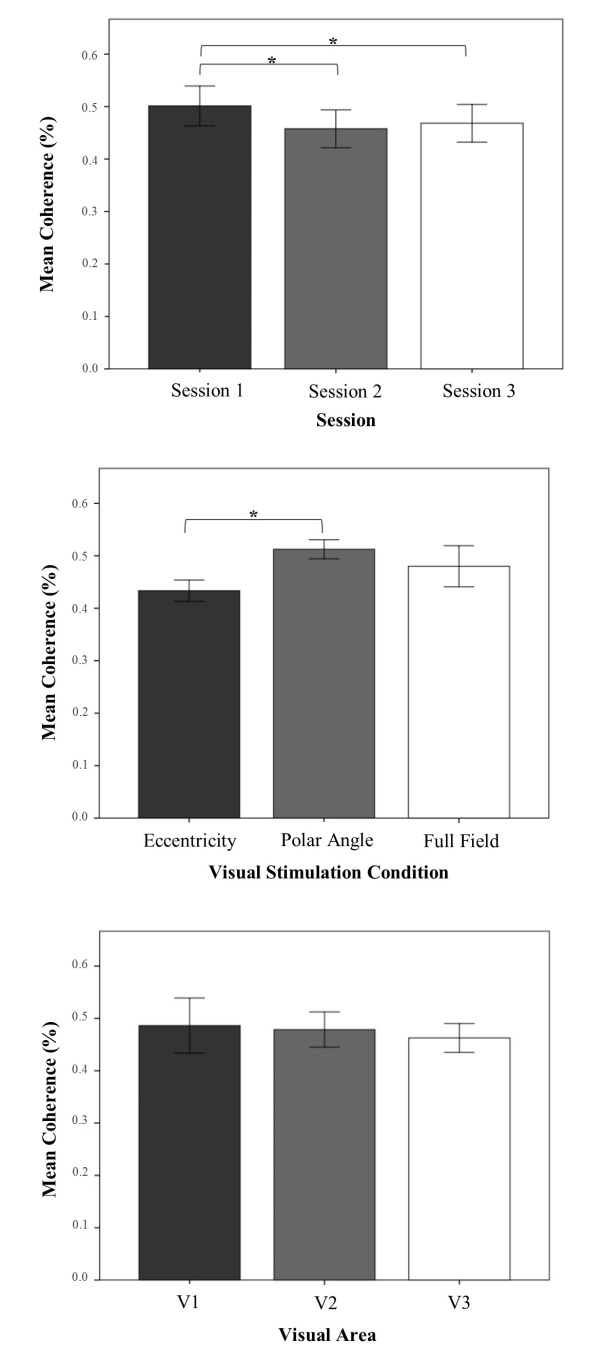


Fig. S8. Quantitative comparison of coherence (re-transformed mean ± SEM of Z-transformed value) across sessions in V1, V2 and V3 for all visual stimulation conditions. **p*<0.05.
